# Staging Breast Cancer with MRI, the T. A Key Role in the Neoadjuvant Setting

**DOI:** 10.3390/cancers14235786

**Published:** 2022-11-24

**Authors:** Camilla Panico, Francesca Ferrara, Ramona Woitek, Anna D’Angelo, Valerio Di Paola, Enida Bufi, Marco Conti, Simone Palma, Stefano Lo Cicero, Giovanni Cimino, Paolo Belli, Riccardo Manfredi

**Affiliations:** 1 Department of Bioimaging, Radiation Oncology and Hematology, UOC of Radiologia, Fondazione Policlinico Universitario A. Gemelli IRCSS, Largo A. Gemelli 8, 00168 Rome, Italy; 2 Institute of Radiology, Catholic University of the Sacred Heart, Largo A. Gemelli 8, 00168 Rome, Italy; 3 Medical Image Analysis and AI (MIAAI), Danube Private University, 3500 Krems, Austria; 4 Department of Radiology, University of Cambridge, Cambridge CB2 0QQ, UK; 5 Cancer Research UK Cambridge Centre, Cambridge CB2 0RE, UK

**Keywords:** early-stage breast cancer, magnetic resonance imaging, neoadjuvant chemotherapy, pathological complete response

## Abstract

**Simple Summary:**

Neoadjuvant chemotherapy (NACT) indications have expanded from inoperable locally advanced to early-stage breast cancer. The pivotal role of magnetic resonance imaging (MRI) with morphological and functional modalities is making headway in the assessment of tumor size in the staging, residual tumor and prediction of response. Radiomics and radiogenomics MRI applications in the setting of the prediction of the response to NACT in breast cancer are continuously increasing. Nevertheless, there are still controversies regarding the indication of MRI in this setting. Given numerous publications and clinical trials regarding this field, we sought to summarize this complex literature to help clarify the role of MRI in evaluating the tumor size in the staging, response assessment, and surgical planning in early-stage breast cancer patients receiving NACT.

**Abstract:**

Breast cancer (BC) is the most common cancer among women worldwide. Neoadjuvant chemotherapy (NACT) indications have expanded from inoperable locally advanced to early-stage breast cancer. Achieving a pathological complete response (pCR) has been proven to be an excellent prognostic marker leading to better disease-free survival (DFS) and overall survival (OS). Although diagnostic accuracy of MRI has been shown repeatedly to be superior to conventional methods in assessing the extent of breast disease there are still controversies regarding the indication of MRI in this setting. We intended to review the complex literature concerning the tumor size in staging, response and surgical planning in patients with early breast cancer receiving NACT, in order to clarify the role of MRI. Morphological and functional MRI techniques are making headway in the assessment of the tumor size in the staging, residual tumor assessment and prediction of response. Radiomics and radiogenomics MRI applications in the setting of the prediction of response to NACT in breast cancer are continuously increasing. Tailored therapy strategies allow considerations of treatment de-escalation in excellent responders and avoiding or at least postponing breast surgery in selected patients.

## 1. Introduction

Breast cancer (BC) is the most common cancer among women worldwide and represents one of the leading causes of mortality and morbidity. In the last decade, the survival rate of BC has been rising thanks to screening and improvements in pharmacological and surgical treatments [1].

Neoadjuvant chemotherapy (NACT) is a systemic treatment administered before surgery [2]. The development of this pharmacological treatment is intended to intervene in patients with locally advanced breast cancer where operability conditions are poor, in order to downstage the tumor and increase operability. Concerns raised by the delayed surgery and the local control after the downstaging of the tumor have been overcome by the results of a randomized clinical trial which showed that the effectiveness of NACT is equivalent to adjuvant chemotherapy in operable breast cancer [3].

NACT is currently also used for patients with early disease, categorized as operable, showing clinically node-negative breast cancer with an unfavorable profile, for whom an adjuvant systemic therapy is recommended [4]. NACT has the ability to reduce the extent of surgical intervention both in breast and axilla. It also improves breast conservative surgery (BCS) and may avoid a complete dissection of the axillary lymphnodes in those patients with a good response. The assessment of early response in vivo is a further advantage of NACT, allowing personalized treatment changes and providing individualized post-treatment informations on prognosis for possible additional adjuvant therapy; this is the case especially for patients with human epidermal growth factor receptor 2 (HER2) positive and triple negative breast cancer [5].

The definition of pathological complete response (pCR) varies; for the purpose of designing trials, the FDA recognizes either the absence of invasive cancer in the breast and in the axillary lymph nodes (ypT0/is N0) or invasive cancer and in situ cancer (ypT0 N0) after NACT [6].

Achieving pCR has been proven to be an excellent prognostic marker indicating better disease-free survival (DFS) and overall survival (OS) compared with patients still showing residual invasive disease [7]. The I-SPY2 trial (in a three-year follow-up analysis) indicates that, regardless of subtype and treatment regimen, achieving pCR after neoadjuvant therapy implies approximately an 80% reduction in recurrence rate [8]. Large trials, such as The National Surgical Adjuvant Breast and Bowel Project B-18 and B-27 clinical trials, comparing DFS and OS in two groups of patients randomized to either neoadjuvant or adjuvant therapy found no significant differences [9]. These results support the use of NACT in patients who meet the indication for adjuvant therapy [10,11].

Breast imaging in the pre-treatment evaluation is mandatory to assess the extent of disease and guide biopsies for confirming pathology. It should include a digital mammogram (optionally with tomosynthesis), breast and axillary hand-held ultrasound (HHUS) and in selected patients, magnetic resonance imaging (MRI). Histopathologic confirmation by core biopsy under imaging-guidance and assessment of estrogen receptor (ER), progesterone receptor (PR), HER2, and ideally Ki-67 must be obtained before initiating treatment [12].

MRI is the most accurate method for assessing tumor size in the staging and response assessment after NACT [13,14]. According to the EUSOMA guidelines, pre-treatment breast MRI should be performed in those individuals potentially operable before the first course of NACT, under the condition that performing MRI does not significantly postpone NACT initiation. Post-NACT breast MRI should preferably be performed two weeks after the last NACT cycle and within two weeks before surgery; any treatment delay caused by the preoperative MRI should not exceed one month [15]. Moreover, multiple studies investigated the role of MRI in predicting treatment response to NACT [16]. Nevertheless, there are still controversies regarding the indication of MRI in this setting.

Given numerous publications and clinical trials in this field, we sought to summarize this complex literature to help clarify the role of MRI in evaluating the tumor size in the staging, response, and surgical planning in early-stage breast cancer receiving NACT.

Relevant papers were found through computerized English-language literature searches of MEDLINE and PubMed Databases in the last 10 years up to May 2022, including the terms: “tumor size”; “breast cancer”; “magnetic resonance imaging”; “neoadjuvant chemotherapy”. In addition, articles were traced through references listed in relevant papers and in previous reviews.

## 2. MRI Accuracy in Assessing Tumor Size Compared to Other Imaging Techniques (HHUS; Mammography; Automated Breast Ultrasound (ABUS); Contrast-Enhanced Mammography (CEM))

The goals of pre-therapy imaging are to determine local-regional staging and to check the contralateral breast. The extent of residual disease, displayed by imaging after NACT, aids in guiding surgical planning.

Data from single-institution and [17,18] multicenter studies [19] validate that baseline MRI is more sensitive in assessing tumor size and detecting multifocal and multicentric cancers than conventional imaging. Under- and overestimations are more likely to be found in non-mass enhancements, rather than in masses [20]. In contrast with initial theories, MRI also holds the potential to detect ductal carcinoma in situ (DCIS) and extensive intraductal spread much better than with mammography [21]. In a prospective study by Kuhl et al. [22], MRI detected intermediate and high-grade DCIS with a sensitivity ranging form 91% to 98%, probably due to significantly higher vessel density than in low grade DCIS. 

A meta-analysis of 44 studies between 1990 and 2008, including 2050 patients, found that MRI after NACT generally had high sensitivity (83–87%) and heterogeneous specificity (54–83%) in detecting residual disease [13]. This meta-analysis adds that the ability of MRI to differentiate residual malignancy from pCR had an overall AUC of 0.88 and that overall accuracy varied according to the definition of pCR and study period. Underestimation of residual disease is more common in non-mass forming lesions than for mass lesions [23]. When using MRI to evaluate the tumor response, some authors suggested that the type of chemotherapy agent should be considered. In HER2 negative patients treated with bevacizumab and paclitaxel [24,25], and in patients treated with taxane-containing regimens, residual disease was frequently undervalued, likely due to antiangiogenic treatment effects limiting tumoral contrast enhancement.

Data about mammography and HHUS in the setting of response to NACT are variable. The initial mammographic appearance of the tumor affects the accuracy of mammography in the evaluation of residual tumor size. Huber et al. [26] found that, for tumors presenting as masses with well circumscribed margins, the correlation of post-therapy tumor size on mammography with histopathology was high (*r* = 0.77). In contrast, masses with ill-defined margins had a lower correlation (*r* = −0.19). Although a decrease in tumor size indicates treatment response, there is no correlation between the change in the extent of the calcifications after NACT and pathologic complete response [27].

Reportedly, HHUS is more accurate than mammography in estimating residual tumors. No difference in accuracy was found between MRI and HHUS, even though HHUS is deemed unsuitable for monitoring tumor size because it is subjective and operator dependent [28]. Few studies have proposed Automated Breast Ultrasound (ABUS) as a valuable tool to monitor patients during NACT and early breast cancer response prediction, as it shows comparable accuracy to MRI [29,30]. When mammography and HHUS found no residual disease, the likelihood of a pathologic complete response was 80% [31]. The use of both imaging modalities improved the accuracy of predicting a pathologic complete response after NACT better than either modality alone [32].

Contrast-enhanced mammography (CEM) allows the evaluation of vascularized lesions such as with breast MRI [33]. Compared with mammography and HHUS, CEM shows greater sensitivity for breast cancer detection without reducing specificity [34]. CEM has higher spatial resolution than MRI revealing details that are approximately ten times smaller [35]. The advantages of CEM over breast MRI are the shorter examination time and lower costs [36]. Furthermore, CEM allows the evaluation of both microcalcifications (on the low-energy image) and enhanced structures (on the recombined image) at the same time, overcoming MRI and its variable sensitivity with in situ cancers [37]. Some studies have shown that CEM seems at least as reliable as MRI in the assessment of response to NACT and may be an alternative if MRI is contraindicated or its availability is limited. MRI still maintains its superiority in cancers located in anatomic areas difficult to depict on CEM (prepectoral, far medial, high up in axillary tail) [38,39].

## 3. Influence of Tumor Biology on MRI Accuracy

It is now generally acknowledged that there are various subtypes of breast cancer, each with a unique pathophysiology, prognosis, and course of treatment. Four primary molecular subtypes of invasive breast cancer have been found by gene expression profiling and are identified in clinical care based on immunohistochemistry: luminal A, luminal B, HER2 positive, and triple negative (TN) [40]. Many studies have examined the correlation between MRI characteristics and tumor subtype even if, as recently stated by a systematic review, the presence of particular features is still only “suggestive” rather than a diagnostic standard [41].

Luminal A is the most prevalent type of breast cancer, commonly managed by surgery and hormone therapy. Even though they are ER-positive, luminal B tumors are more resistant to endocrine therapy, having in common many molecular characteristics with ER-negative subtypes [42]. Luminal B breast cancers are more aggressive than luminal A breast cancers, with lower DFS rates and higher tumor necrosis [43]. Luminal A and luminal B exhibit very similar MRI features; the heterogenous enhancement in luminal B tumors may be indicative of tumor necrosis or neo-angiogenesis that is related to abundant, fibroblast growth factor receptor [44]. Multicentricity, multifocality, skin and nipple-areolar involvement, and axillary disease are further characteristics associated with luminal B cancers [45].

HER2 positive cancers have several MRI characteristics that are comparable to those of luminal tumors; heterogeneous non-mass enhancement, perilesional oedema, and higher apparent diffusion coefficient (ADC) values than other subtypes, are a few characteristics that may indicate HER2 positive cancers [46].

On MRI, TN breast cancer is the most distinct tumor. Masses with smooth margins, round or oval shape, high T2 signal intensity, rim enhancement, and intratumoral necrosis are suggestive of histopathological TN breast cancer [47,48].

Similar MRI appearance can be seen in uncommon histological forms of invasive breast cancers, representing a diagnostic challenge, e.g., mucinous carcinoma that exhibits high T2 signal intensity due to its predominant mucin component, but it often shows a persistent type of curve [49].

Enhancement kinetics showed poor predictability of molecular subtypes. Poor prognosis predictors are early maximum enhancement, wash-out in the delayed phase and rim enhancement and they are related with higher histologic grade, positive Ki-67, and negative ER status [50].

A correlation was found between the type II enhancement curve and luminal A tumors, possibly explained by limited neo-angiogenesis compared to other molecular subtypes [51]. Table 1 provides the main features of breast cancer molecular subtype.

MRI accuracy has different discrepancy according to histological subtype; data indicate that infiltrating ductal carcinoma has a significantly lower mean discrepancy when compared with infiltrating lobular carcinoma [52].

## 4. Early Prediction of Pathological Outcome after NACT

Early prediction of pCR following NACT could be an important tool for personalized medicine, allowing the selection of patients eligible for NACT [53].

Many studies have demonstrated how the evaluation of multiparametric breast MRI obtained before the start of NACT can predict which cancer will achieve pCR, through the analysis of morphological and functional features and the application of advanced imaging and artificial intelligence (AI) techniques.

Thus, the challenge remains the early and accurate predictions of NACT response.

### 4.1. Morphological Characteristics

Among the morphologic MRI features, an expansively growing carcinoma forming a relatively well-defined round/oval or lobulated mass is often associated with low rates of pCR, suggesting that careful attention should be paid during the course of the therapy in these types of tumors [54]. In patients with HER2 positive or TN breast cancer, one recent study identified an orientation of growth parallel to Cooper’s ligaments as an accurate predictor of pCR [55]. Indeed, the growth pattern may be a sign of a tumor’s microarchitecture and influence on its micro-environment: tumors growing more slowly can cause desmoplastic reaction and grow crossing Cooper’s ligaments, while tumors with faster and more expansive growth will propagate parallel to Cooper’s ligaments [55].

In a study by Thompson et al. [56], the authors found a significant association between tumor spread at baseline MRI and pCR, with multicentric tumors being associated with a lower possibility of pCR compared to multifocal or single lesion. Likewise, multifocal multicentric lesions and non-mass enhancement in pre-treatment MRI were significantly associated with absence of pCR in a multivariate analysis of radiological findings by Choi et al. [57].

### 4.2. Background Parenchyma Enhancement (BPE)

Some authors have suggested a correlation between the background parenchymal enhancement (BPE) and tumor response after NACT. BPE is defined as normal enhancement of the fibro-glandular tissue on contrast-enhanced (CE) MRI and is thought to be associated with poorer prognosis: several studies found a significant association between high BPE on baseline MRI and worse recurrence free survival (RFS) [58,59]. This is probably due to higher perfusion into the breast, leading to higher BPE, and proangiogenic breast ecosystem for tumor growth or higher amounts of physiologically active breast tissue more susceptible to tumor transformation [60]. Nevertheless, evaluation of the BPE of the tissue surrounding the tumor can be difficult and relies on the selection of a proper image and the placement of the region of interest.

In a study by Preibsch H et al. [61], the authors found that the reduction of BPE on CE-MRI in patients undergoing NACT may predict tumor response. Since CE-MRI is performed bilaterally, some authors measured the BPE of the contralateral healthy breast and found no associations between BPE at pre-treatment MRI and response to NACT [62,63]. Thus, suggesting that other factors such as Ki-67 or menopausal state play a more important prognostic role.

### 4.3. Diffusion Weighted Imaging (DWI) and Apparent Diffusion Coefficient (ADC)

Functional imaging sequences, such as DWI-MRI and CE-MRI, allow the analysis of biological characteristics of tumors, such as cellularity and neo-angiogenesis, by assessing cellular density of a tissue, quantified using ADC map. However, the analysis of the ADC value on pretreatment and pCR imaging remains controversial, since some tumors may have low cellularity (and high ADC value) due to local necrosis secondary to hypoxia or local fibrosis or desmoplastic components [64]. According to some authors, a tumor with low cellularity is less likely to respond to NACT, possibly due to lack of perfusion and/or slow tumor growth that causes impaired drug administration; while highly cellular tumors (therefore with low ADC values) are more chemo-sensitive [55,65,66].

In one study, the best pre-treatment ADC cut-off value to distinguish between responders and non-responders was found to be 0.55 mm^2^/s based on the ROC curve analysis (area under the ROC curve was 0.65; 95% CI, 0.415–0.831) [55]. In another study the best pretreatment ADC cutoff was 1.17 × 10^−3^ mm^2^/s using a high *b*-value of 750 s/mm^2^, with a sensitivity of 94% and a specificity of 71% [66].

Conversely, some other authors found no significant difference in the mean ADC value between responders and non-responders in the general population [67,68]. However, when performing the analysis into different subgroups according to tumor phenotypes, the authors observed a statistically significant difference in the mean value of ADC between responders and non-responders in TN and HER2 positive tumors [68,69]. The difference in results indicate an effect modification according to the classified subtype.

Some works investigated total choline (TCho) levels in breast cancer through Magnetic Resonance Spectroscopy (MRS). TCho is a biomarker of elevated cellular turnover and is thought to be an early imaging predictor of response to treatment but its prognostic value remains unconfirmed [70,71]. However, the technical complexity and limited diffusion of MRS inhibit its use for response assessment in multicentric studies [72].

### 4.4. Triple Negative Breast Cancer

Different cancer subtypes show different pCR rates with higher rates in HER2 positive and TN breast cancer [73], reaching 68–80% in patients having carboplatin or dual HER2 blockade [74].

However, TN breast cancer is more aggressive than other subtypes and associated with a higher rate of relapse and a lower rate of OS [65,71].

TN breast cancer is more likely to display intramammary edema, resulting from lymphovascular invasion. Peritumoral edema occurs due to local reaction of the surrounding tissue to the tumor, pre-pectoral edema is determined by marked lymphovascular invasion, whereas subcutaneous edema reflects the lymphovascular invasion. The correlation between response to NACT and the presence of edema is controversial. Bae et al. [75] revealed that the presence of peritumoral edema is associated with low pCR, even though it isn’t significantly associated with worse RFS. However, more recent studies found no correlation between the presence of intramammary edema and response to NACT [76,77]. Reported edema might not imply the reporting of the predominant underlying cause (inflammation, angiogenesis, infiltration of tumor cells, tumor emboli) and this may explain the difference of the findings in the literature.

Another common finding in TN breast cancer is intratumoral necrosis, probably due to the increased mitotic activity, which is often associated with poor or no response to NACT [78]. However, a recent study found no significant correlation between the presence of intratumoral necrosis and pCR in patients with TN breast cancer [77]. Small numbers of patients might affect these conflicting findings.

In addition, it has been shown that TN breast cancer presenting as an irregular mass is less likely to respond to NACT than that with other appearances [78].

In a study by Li et al. [79], a nomogram based on baseline dynamic CE-MRI was developed to predict pCR; in particular, it was observed that time to peak (TTP), tumor volume measured on CE-MRI and androgen receptor (AR) grade were independent predictors of pCR.

### 4.5. What’s New (Radiomics, Machine Learning and Radiogenomics)

Radiomic analysis is a method to extract quantitative information from each voxel of radiological images, to predict clinical data. Radiomics applications are constantly increasing given their ability in the prediction of pCR in various cancer types, including breast cancer [80]. In a retrospective study, Cain et al. [81], developed multivariate machine learning models based on pre-treatment MRI features that were able to predict pCR in TN and HER2 positive patients. Similarly, Liu et al. [82] developed and validated radiomics models combining T2-w imaging, DWI, and contrast-enhanced T1-w imaging on pre-treatment MRI. The radiomic signature performed relatively well in ER-positive, HER2 negative and TN groups. Braman et al. [83] examined intratumoral and peritumoral features, since they both contribute to response predictions. Recently, Huang et al. [84] have demonstrated that the tumor shrinkage pattern can be accurately predicted by a model that combines clinicopathologic appearances and radiomics features. These findings imply that pre-treatment breast MRI could be a means to stratify patients and address them to more appropriate treatment options, tailoring treatment and thus improving quality of life. Radiogenomics associates radiomics imaging features to tumor genetic profiles and represents an exciting field of research that might further improve the performance of predictive models [85,86].

## 5. The Role of MRI in the Preoperative Assessment of Residual Disease and Pathological Complete Response (pCR)

As stated before, the definition of pCR is still the object of debate with some trials defining it as the absence of both in situ and invasive cancer while others considering only the invasive component [87]. The accuracy of MRI in detecting residual disease after NACT differs according to pCR definition and it is higher when the resolution of invasive disease only is considered pCR [13,88].

pCR has been proven to be a good prognostic marker to predict long-term survival in breast cancer [7,89]; therefore, it is considered a suitable surrogate end point for patients with luminal B/HER2 negative, HER2 positive (non-luminal), and TN disease but not for those with luminal B/HER2 positive or luminal A tumors [86,90]. However, only about 30% of patients achieve pCR after NACT, and the pCR rate changes according to different molecular subtypes, as for tumor size and treatment regimen [91].

In order to reach the maximum surgical advantage from NACT, it is crucial to correctly evaluate the tumor response and residual disease prior to surgery. The Response Evaluation Criteria in Solid Tumors (RECIST) 1.1 guidelines [92] recommend measurement of the longest diameter of a solid tumor in at least one dimension. Multicentric disease should be evaluated by summing the largest diameter of the detectable tumors. Although MRI can both over- and underestimate residual disease [13], the longest diameter measured on the first contrast-enhanced phase of MRI performed after NACT better evaluate the extent of residual disease than clinical exam, mammography, and HHUS [14,17,93].

### 5.1. Pattern of Tumor Response

**Figure 1:**
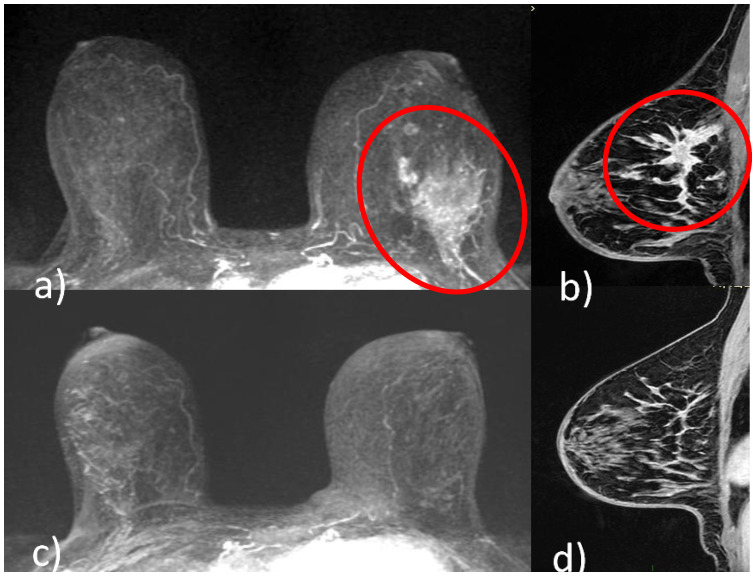
A 51-year-old patient with invasive ductal carcinoma (ER 40% PR 3% Ki67 40% HER2 3+) of the upper outer quadrant of the left breast. Pre-NACT CE-MRI revealed an area of non-mass enhancement with segmental distribution in the left breast that extends to pectoralis muscle without signs of invasion (red circle, (**a**) axial maximum intensity projection reconstruction image; red circle, (**b**), sagittal post-contrast T1-weighted image). At the end of NACT, CE-MRI showed no residual tumor (pCR) ((**c**) axial maximum intensity projection reconstruction image; (**d**) sagittal post-contrast T1-weighted image).

**Figure 2:**
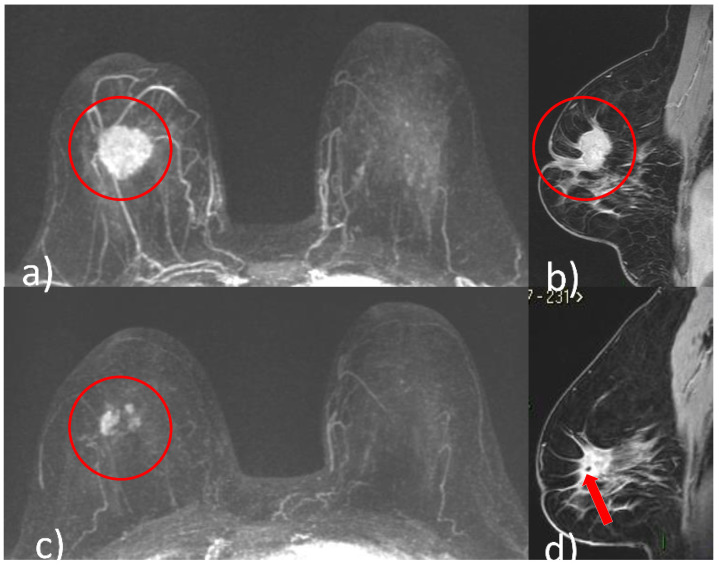
A 48-year-old patient with G2, luminal B, HER2 positive right breast cancer undergoing NACT. Pre-treatment breast CE-MRI showed an oval mass with irregular margins, nipple invasion and skin retraction at the junction of upper quadrants of the right breast (red circle, (**a**) axial maximum intensity projection reconstruction image) (red circle, (**b**) sagittal post-contrast T1-weighted image). After NACT, multifocal shrinkage was depicted by CE-MRI (red circle (**c**), axial maximum intensity projection reconstruction image). (**d**) Signal void artifact caused by tissue marker clip inside the residual mass is well visible on the sagittal T1-weighted post-contrast image (red arrow).

Different shrinkage patterns are seen in breast cancer after NACT, including no residual tumor (pCR) (Figure 1), concentric shrinkage (i.e., only one residual invasive tumor focus, without DCIS), multifocal shrinkage (i.e., more than two invasive) (Figure 2), diffuse shrinkage (i.e., a main residual invasive focus with surrounding satellite DCIS), stable disease (SD), and progressive disease (PD) [84].

Kim et al. [94] found that the extent of residual disease obtained at MRI was significantly correlated with the evaluation obtained at pathology, excluding crumbling/residual multicentric shrinkage pattern. Tumor response as multiple, scattered deposits may also make assessment of the longest diameter difficult, with different approaches to measurement that either include [95] or exclude [96] intervening normal tissue. Concentric shrinkage or multicentric shrinkage patterns halfway through therapy often indicate response at the end of therapy, while diffuse non-mass enhancement, stable disease, and progression are strongly related with non-response [97]. The probability of underestimating residual disease is reported as being higher for non-mass enhancement than for masses. Reactive inflammation, fibrosis or necrosis in response to NACT may appear as areas of enhancement on MRI, that might be challenging to distinguish from residual tumor [18,98].

Some authors tried to quantify the degree of residual enhancement by using the lesion to background parenchymal signal enhancement ratio (SER), which has been found to increase the specificity in detecting residual tumor rather than using size criteria alone (with SER ≤ 1.6 indicating pCR) [99].

It has been suggested that the type of chemotherapy agent should be considered when using MRI in the evaluation of tumor response. Patients treated with taxane-containing regimens [23] and in HER2 negative patients receiving bevacizumab or paclitaxel [24,25], residual disease was frequently underestimated.

### 5.2. Impact on Therapy

MRI also allows in vivo evaluation of treatment efficacy, which may permit changing the treatment approach if the tumor is not responding. Some studies reported the ability of MRI to assess the efficacy of treatment early, after 1–2 cycles of NACT, based on imaging data of volumetric changes, kinetic analysis, both [100] and metabolic changes [101,102]. However, the use of MRI to inform changes to the NACT regimen has been shown only in one retrospective study [103].

Overall, based on its ability to show the extent of tumor after NACT and increasing numbers of studies demonstrating early response prediction, MRI is a useful tool in helping the multidisciplinary team to choose the most suitable therapeutic planning, especially surgical treatment aimed at local disease control. However, up to now, no randomized control trial has demonstrated that using MRI for monitoring the response to NACT increases the rate of breast conserving surgery. On the other hand, also in the neoadjuvant setting, MRI is considered useful for appropriate surgical decision making likewise to preoperative MRI in the absence of NACT [15].

## 6. Surgical Planning

Breast conserving surgery (BCS) is nowadays the standard surgical treatment for most early-stage breast cancers (EBC) and locally advanced breast cancers after good NACT response [4,6,104,105]. EBC patients ineligible for BCS at the time of diagnosis, may profit from NACT to decrease tumor size facilitating BCS [106]. To achieve BCS, pCR is not a precondition, but the better the response to treatment the higher the chances of successful BCS [107]. BCS is the desirable alternative to mastectomy in tumors up to 2 cm [15], as it has been shown that there is no significant difference in mortality rates among the two treatment options, with however, a 10 year recurrence rate between 5 and 10% in the case of BCS and radiation therapy [108,109,110].

Although the diagnostic accuracy of MRI has been shown repeatedly to be better to conventional examinations in assessing the extent of breast disease, literature does not agree that the routine use of MRI, as part of pre-operative staging even after NACT, leads to an improvement in surgical outcomes. For this reason, most guidelines and recommendations suggest that preoperative MRI should only be used in selected cases [109,111,112,113,114].

The fear that MRI may lead to an increase in the rate of mastectomies, beyond what is necessary, has not yet been completely dispelled [115].

However, in recent years, studies with large numbers of patients have shown that a small increase in mastectomy rates can be well compensated by a reduction in recurrences; for example, Sardanelli et al. [112] performed a prospective multicenter study with two cohorts of patients with breast cancer: one undergoing conventional imaging before surgery and the other group undergoing additional MRI for local staging; patients of MRI group had 4.4 OR of getting mastectomy compared to the group that did not perform, but this was offset by a lower reoperation rate (3%).

Not many studies have evaluated the long-term outcome of presurgical MRI, and most studies do not indicate a decrease in metachronous second breast tumors [21]. There is no evidence for improved DFS or OS because of presurgical MRI, but evidence suggest that early identification of second cancers increase overall survival [111].

In patients with excellent response to NACT and subsequent pCR, it is oncologically safe to de-escalate treatment [74,116,117]. Accurate tailoring of NACT allows consideration of individualized locoregional treatment strategies. Heil et al. [118] put it to the extreme: they proposed to postpone or avoid breast surgery, trying a “watch and see” strategy, in patients that showed pCR at the end of NACT. The same approach is recently being used also in excellent responders to NACT in different types of cancer like rectal and esophageal: unless local recurrence is found, surgery is not performed [119]. There is no evidence consensus concerning the effect of misdiagnosed NACT minimal residual disease [120,121]. Consequences could vary depending on whether there is an in situ or invasive disease, lymphovascular invasion, and on the amount of residual disease. Adjuvant treatment (anti-HER2 or hormonal therapies) or radiation treatment, the current standard approach even in pCR patients, could be used to control misdiagnosed minimal residual disease. According to recent studies, in triple-negative and HER2 positive patients with residual disease after NACT (confirmed at pathological examination), supplementary systemic therapy with capecitabine and T-DM1 may improve overall survival and decrease the recurrence ratio [74,116].

The data show that patients with breast cancer and pCR to NACT are very unlikely to face distant recurrence; in contrast, minimal residual pathology with subsequent risk of local relapse is still possible. Studies are currently underway to identify pCR, using barely invasive biopsies in women who have had partial/complete radiologic response after treatment, in order to stratify low-risk patients [122].

In addition, there may also be subset of patients who do not benefit from surgery after treatment; however, there is currently insufficient scientific evidence. Therefore, several questions remain unsolved, and it remains a great challenge for the future to conduct high-quality clinical trials that can answer these questions [117].

## 7. Conclusions

We are aware of the fact that this narrative review might have some limitations: it is non-systematic and only English language papers have been considered. Moreover, the rapid information produced in the literature on this topic might have caused some missing new relevant paper. However, the overall evaluation points out that indications for neoadjuvant systemic treatment have growth from inoperable locally advanced to early-stage breast cancer. MRI plays a pivotal role and with morphological and functional modalities is making headway in tumor size evaluation, in the staging and in response prediction. Radiomics and radiogenomics MRI applications in the setting of breast cancer NACT response prediction are continuously increasing. Tailored systemic therapy strategies allow considerations of treatment de-escalation in excellent responders and avoiding or at least postponing breast surgery in selected patients.

## Figures and Tables

**Table 1:** MRI main features of breast cancer molecular subtype.

	Shape and Margin	T2 Signal	Enhancement Pattern	Others
Luminal A	Irregular, spiculated	Low/iso	Heterogenous	-
Luminal B	Irregular, not circumscribed	Low/iso	Heterogenous	Multifocal, multicentric, skin and/or nipple invasion
HER2 positive	Irregular, not circumscribed	Low	Heterogenous	Non-mass enhancement, peritumoral oedema, tumor necrosis
Triple Negative	Round/oval, circumscribed	High	Rim enhancement	Peritumoral oedema, tumor necrosis
